# MODE EFFECTS ON COGNITIVE FUNCTIONING ASSESSMENTS IN THE HEALTH AND RETIREMENT STUDY

**DOI:** 10.1093/geroni/igac059.652

**Published:** 2022-12-20

**Authors:** Jessica Faul, Ben Domingue, Ben Stenhaug, Brady West, Kenneth Langa, David Weir

**Affiliations:** University of Michigan, Ann Arbor, Michigan, United States; Stanford, Stanford, California, United States; Stanford, Sanford, California, United States; University of Michigan, Ann Arbor, Michigan, United States; University of Michigan, Ann Arbor, Michigan, United States; University of Michigan, Ann Arbor, Michigan, United States

## Abstract

As the population of the US ages, there is interest in assessing health conditions associated with age and longevity, such as age-related decline in cognitive functioning. As a result, there is an increased focus on measuring cognitive functioning in surveys of older populations. One challenge relates to conducting comparable measurement across survey modes (e.g., phone vs. web). Compounding this is that mode of survey administration is often not assigned randomly making inter-group comparison more difficult. This paper addresses these issues using a novel experiment embedded within the Health and Retirement Study (HRS). The HRS, a US-based cohort of people over 50, has measured cognition since its inception using both in-person and telephone modes. In 2018, a sample of approximately 3700 respondents was identified as web-eligible based on a prior report of internet access along with other selection criteria. Of these, 60% were randomly selected for the web sample with the remainder serving as controls, assigned to telephone mode for comparison purposes. We deploy techniques from item response theory (IRT) and differential item functioning (DIF) to estimate the difference in cognitive functioning between web and phone respondents in 2018 based on longitudinal cognition data collected prior to 2018. Second, we estimate the overall effect of taking the survey via the web as compared to the phone. Third, we examine item-level variation in the magnitude of the mode effect and suggest possible methods for adjustment to support longitudinal consistency. These results are important in guiding future research that utilizes web-based cognitive measures.

